# Supplementary material for: The application of large language models in bariatric surgery: A scoping review
Source: PLoS One. 2026 Jun 5;21(6):e0350748. doi: 10.1371/journal.pone.0350748 (PMC13240874; doi:10.1371/journal.pone.0350748)
Supplement: S1 Checklist — (DOCX) [file pone.0350748.s001.docx]

**Preferred Reporting Items for Systematic reviews and Meta-Analyses extension for Scoping Reviews (PRISMA-ScR) Checklist**

| **SECTION** | **ITEM** | **PRISMA-ScR CHECKLIST ITEM** | **REPORTED ON PAGE #** |
| --- | --- | --- | --- |
| **TITLE** | | | |
| Title | 1 | Identify the report as a scoping review. | The Application of Large Language Models in Bariatric Surgery: A Scoping Review |
| **ABSTRACT** | | | |
| Structured summary | 2 | Provide a structured summary that includes (as applicable): background, objectives, eligibility criteria, sources of evidence, charting methods, results, and conclusions that relate to the review questions and objectives. | **Abstract**  **Background:**Exploratory applications of large language models within the specialized field of metabolic and bariatric surgery have begun to emerge. Nevertheless, existing research remains fragmented, lacking comprehensive integration.  **Objective:**To conduct a scoping review of studies on the application of large language models in the field of metabolic and bariatric surgery, aiming to provide a reference for clinical practice and future research.  **Methods:**This scoping review adhered to the Joanna Briggs Institute methodological framework and followed the preferred reporting items for systematic reviews and meta-Analyses extension for ccoping reviews (PRISMA-ScR) guidelines.PubMed, Web of Science, The Cochrane Library, Embase, CINAHL, CNKI, Wanfang, and VIP databases were searched for relevant studies, with the search timeframe from database inception to November 2025. The included literature was summarized and analyzed.  **Results:**A total of 21 English-language studies were included. LLMs were primarily applied in scenarios such as patient education and information consultation, clinical decision support, and professional knowledge assessment. While LLMs performed well in information-provision tasks, they showed low consistency with expert opinions in complex clinical tasks such as individualized surgical recommendations. Performance varied across different models, with GPT-4 generally demonstrating superior performance, and domain-specific models showing professional potential. Current research still faces challenges regarding information accuracy, readability, and clinical applicability.  **Conclusion:**Large language models hold auxiliary potential in the field of metabolic and bariatric surgery, particularly for knowledge dissemination and patient education. However, their reliability in complex clinical decision-making remains limited. Future efforts should focus on conducting high-quality studies, advancing model specialization and standardized evaluation, and exploring safe and effective human-AI collaboration models.  **Keyword:**Bariatric surgery; Metabolic surgery; Metabolic and bariatric surgery; Large language model; Generative artificial intelligence; Scope review. |
| **INTRODUCTION** | | | |
| Rationale | 3 | Describe the rationale for the review in the context of what is already known. Explain why the review questions/objectives lend themselves to a scoping review approach. | The global prevalence of obesity continues to rise.Metabolic and bariatric surgery (MBS) has become an effective intervention for severe obesity, demonstrating not only significant weight loss but also improvement in metabolic parameters and a reduction in the incidence of obesity-related diseases. It is currently one of the most effective approaches for treating severe obesity and related metabolic disorders.However, alongside the sustained growth in surgical volume and the ongoing standardization of the specialty, metabolic and bariatric surgery continues to face a series of clinical challenges and practical pressures.In recent years, the rapid advancement of artificial intelligence (AI) technology, particularly represented by Large Language Models (LLMs), offers a new perspective and potential to address these challenges.Against this backdrop, exploratory applications of LLMs within the specialized field of metabolic and bariatric surgery have begun to emerge. Nevertheless, existing research remains fragmented, lacking comprehensive integration in terms of coverage across different application scenarios, systematic comparison of different model performances, and synthesis of common challenges and future directions faced by the technology. Therefore, this study aims to systematically review the relevant research on the application of LLMs in the field of metabolic and bariatric surgery through a scoping review methodology. |
| Objectives | 4 | Provide an explicit statement of the questions and objectives being addressed with reference to their key elements (e.g., population or participants, concepts, and context) or other relevant key elements used to conceptualize the review questions and/or objectives. | ①In which specific scenarios of bariatric surgery are LLMs applied, and how do they perform?② What are the differences in performance among different LLM models when applied in bariatric surgery? ③ What evidence do existing studies provide regarding the effectiveness of LLMs in bariatric surgery applications? ④What are the main challenges currently faced in these applications, and what are the future directions for development? |
| **METHODS** | | | |
| Protocol and registration | 5 | Indicate whether a review protocol exists; state if and where it can be accessed (e.g., a Web address); and if available, provide registration information, including the registration number. | This review adhered to PRISMA-ScR guidelines. While a formal protocol was not registered, the methodology (including search strategy, inclusion criteria, and data extraction steps) was predefined and reviewed by all co-authors to ensure rigor. Full methodological details are provided in the *Methods* section. Additional documentation is available upon request. |
| Eligibility criteria | 6 | Specify characteristics of the sources of evidence used as eligibility criteria (e.g., years considered, language, and publication status), and provide a rationale. | Inclusion criteria were determined according to the PCC (population, concept, context) principles：①Participants (P): Involving clinical practice, patient management, or medical education in the field of metabolic and bariatric surgery;② Concept (C): The core of the study involves the application of Large Language Models (LLMs), including but not limited to their development, deployment, evaluation, or comparison. Application forms include answering patient inquiries, generating educational materials, providing clinical decision support, etc.;③ Context (C): The application scenario is explicitly limited to metabolic and bariatric surgery. Study types are limited to original research such as quantitative studies, qualitative studies, and mixed-methods studies.Exclusion criteria: ①Study content not directly related to bariatric surgery or the application of LLMs;② Conference abstracts, commentaries, editorials, systematic reviews, literature reviews, case reports, study protocols, guidelines, or consensus statements;③Literature for which the full text is unavailable, data cannot be extracted, or is non-peer-reviewed;④ Literature not published in Chinese or English.Literature published up to November 2025 was searched.  . |
| Information sources* | 7 | Describe all information sources in the search (e.g., databases with dates of coverage and contact with authors to identify additional sources), as well as the date the most recent search was executed. | PubMed, Web of Science, The Cochrane Library, Embase, CINAHL, CNKI, Wanfang, and VIP databases were searched for relevant studies, with the search timeframe from database inception to November 2025. |
| Search | 8 | Present the full electronic search strategy for at least 1 database, including any limits used, such that it could be repeated. | Take PubMed for example:((Large Language Models [Mesh] OR Artificial Intelligence[Mesh]) OR (Generative Pre-trained Transformer[tiab] OR ChatGPT[tiab] OR GPT[tiab] OR (Large[tiab] AND Language[tiab] AND Model[tiab]) OR LLM[tiab] OR LLMs[tiab] OR Gemini[tiab] OR DeepSeek[tiab] OR Copilot[tiab] OR LaMDA[tiab] OR LLaMA[tiab] OR BatGPT[tiab] OR Moss[tiab] OR Sora[tiab] OR Kimi[tiab] OR Ernie Bot[tiab] OR Qwen*[tiab] OR iFLYTEK Spark[tiab] OR ChatGLM*[tiab] OR Bard[tiab] OR Claude[tiab] OR Bing[tiab] OR Perplexity[tiab] OR mistral[tiab] OR Grok[tiab] OR PaLM[tiab] OR Chatsonic[tiab] OR Jasper[tiab] OR Generative Artificial Intelligence[tiab] OR Generative AI[tiab] OR Chatbot[tiab])) AND ((Bariatric Surgery[Mesh] OR Obesity, Morbid[Mesh]) OR (Bariatric[tiab] OR Metabolic Surgery[tiab] OR Weight Loss Surgery[tiab] OR Gastric Bypass[tiab] OR Sleeve Gastrectomy[tiab] OR Gastric Sleeve[tiab] OR Gastric Banding[tiab] OR Duodenal Switch[tiab])) |
| Selection of sources of evidence† | 9 | State the process for selecting sources of evidence (i.e., screening and eligibility) included in the scoping review. | The selection form was developed based on the PCC (Participants, Concept, Context) framework to standardize the inclusion and exclusion criteria.The form also captured author, year, country, tool(s), application scenario category , specific application description, and main findings.To ensure clarity and consistency, the form was pilot-tested on 20 randomly selected articles from the initial search results.Prior to full screening, a calibration exercise was conducted to enhance inter-reviewer agreement. Two independent reviewers screened the same 50 articles using the revised form. Discrepancies were discussed in a consensus meeting, and the form was further adjusted to address ambiguities.  Two reviewers independently screened titles and abstracts of 1,130 identified records after duplicate removal. Articles meeting the PCC criteria advanced to full-text review. For the 52 full-text articles assessed, disagreements were resolved through discussion or consultation with a third reviewer. |
| Data charting process‡ | 10 | Describe the methods of charting data from the included sources of evidence (e.g., calibrated forms or forms that have been tested by the team before their use, and whether data charting was done independently or in duplicate) and any processes for obtaining and confirming data from investigators. | Ningjing Guo and Xuyan Li jointly developed a data chart table and determined to extract the following variables:author, year, country, tool(s), application scenario category , specific application description, and main findings were all involved in the data extraction work. Ningjing Guo and Xuyan Li independently drew data charts and discussed the results using Word. |
| Data items | 11 | List and define all variables for which data were sought and any assumptions and simplifications made. | “We abstracted data on article characteristics (e.g., author, year, country),tool(s) (e.g.,ChatGPT, DeepSeek,Gemini, Copilot,Bing, Bard), application scenario category(e.g.,patient education & information,knowledge Assessment & Exams,clinical decision support,domain-specific Model Development,medical image Processing) specific application description, and main findings .” |
| Critical appraisal of individual sources of evidence§ | 12 | If done, provide a rationale for conducting a critical appraisal of included sources of evidence; describe the methods used and how this information was used in any data synthesis (if appropriate). | This scoping review did not conduct a critical appraisal of individual sources of evidence. The rationale for omitting this step aligns with the methodological framework of scoping reviews, which prioritize mapping the breadth and scope of existing literature rather than synthesizing evidence for clinical decision-making or evaluating methodological quality. The Joanna Briggs Institute (JBI) guidelines for scoping reviews explicitly state that critical appraisal is optional and context-dependent, particularly when the review aims to identify knowledge gaps or characterize interventions rather than assess efficacy or risk of bias.  In this study, this study aims to systematically review the relevant research on the application of LLMs in the field of metabolic and bariatric surgery. The inclusion criteria (PCC framework) emphasized relevance to the application of Large Language Models in bariatric surgery rather than methodological rigor. While the PRISMA-ScR checklist guided reporting, it does not mandate quality assessment for scoping reviews. |
| Synthesis of results | 13 | Describe the methods of handling and summarizing the data that were charted. | The purpose of this research project is to systematically review the relevant research on the application of LLMs in the field of metabolic and bariatric surgery. The main characteristics of the included papers are summarized in Table 2. |
| **RESULTS** | | | |
| Selection of sources of evidence | 14 | Give numbers of sources of evidence screened, assessed for eligibility, and included in the review, with reasons for exclusions at each stage, ideally using a flow diagram. | 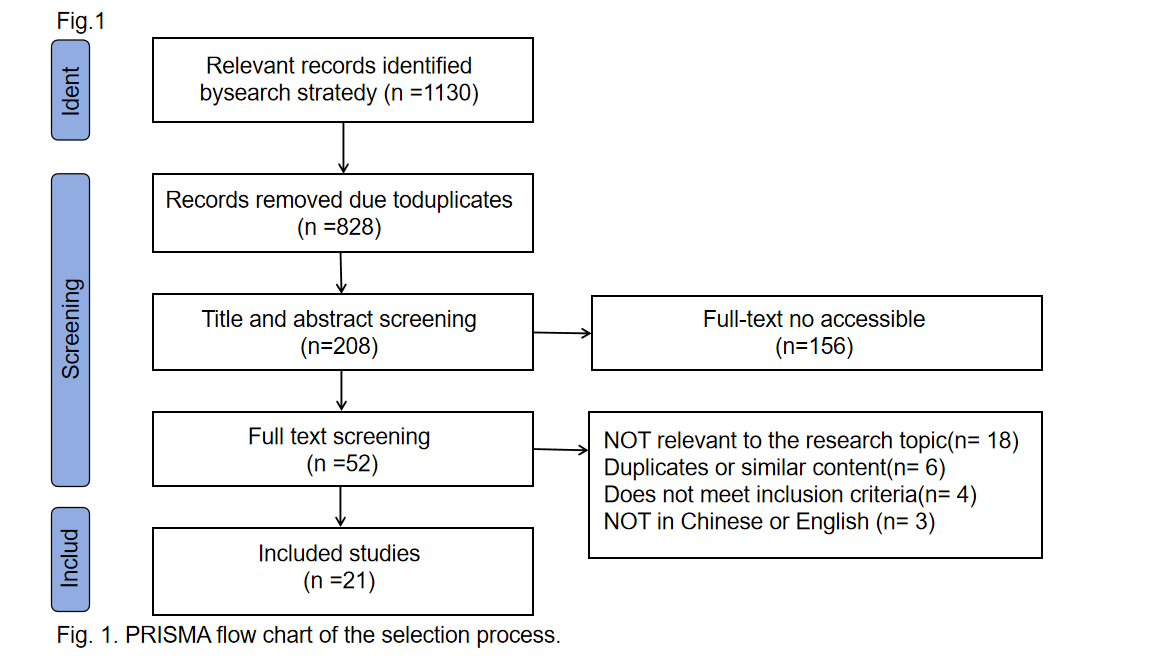 |
| Characteristics of sources of evidence | 15 | For each source of evidence, present characteristics for which data were charted and provide the citations. | The main article characteristics(e.g., author, year, country),tool(s) (e.g.,ChatGPT, DeepSeek,Gemini, Copilot,Bing, Bard), application scenario category(e.g.,patient education & information,knowledge Assessment & Exams,clinical decision support,domain-specific Model Development,medical image Processing) specific application description, and main findings) of the included papers are summarized in Table 2. |
| Critical appraisal within sources of evidence | 16 | If done, present data on critical appraisal of included sources of evidence (see item 12). | This scoping review did not conduct a critical appraisal of individual sources of evidence. |
| Results of individual sources of evidence | 17 | For each included source of evidence, present the relevant data that were charted that relate to the review questions and objectives. | 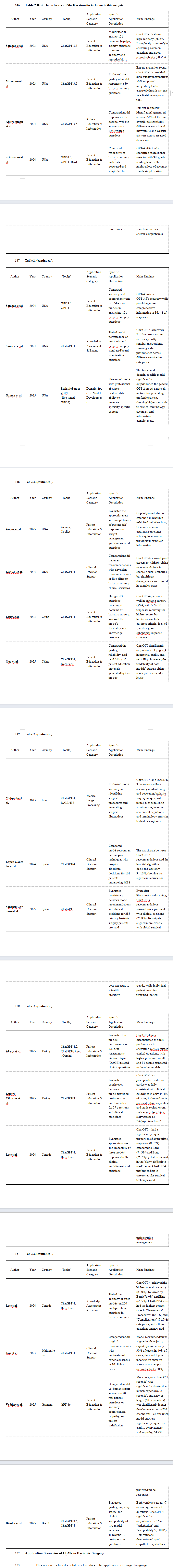 |
| Synthesis of results | 18 | Summarize and/or present the charting results as they relate to the review questions and objectives. | The review of 21 studies on Large Language Models (LLMs) in bariatric surgery reveals their primary application in patient education and information consultation, where they are used for knowledge Q&A and generating educational materials to improve patient engagement. Other explored areas include clinical decision support for surgical recommendations, though consistency with guidelines remains low, and professional knowledge assessment, where models perform well on standardized tests but struggle with clinical reasoning. Additional niche applications in medical image processing and the development of domain-specific models (e.g., BariatricSurgery GPT) show potential but are limited by anatomical inaccuracies or are still in early stages. Performance varies significantly across models, with GPT-4 generally offering higher accuracy than GPT-3.5 or Gemini, while domain-specific models excel in professional terminology. Evaluations indicate that while LLMs achieve high accuracy (up to 86.8%) in common knowledge tasks and offer high efficiency and patient satisfaction regarding empathy, they face challenges in clinical decision-making (expert agreement often below 40%), readability (frequently exceeding recommended grade levels), and information transparency, such as missing citations |
| **DISCUSSION** | | | |
| Summary of evidence | 19 | Summarize the main results (including an overview of concepts, themes, and types of evidence available), link to the review questions and objectives, and consider the relevance to key groups. | Current research reveals a clear dichotomy in the performance of Large Language Models (LLMs) within bariatric surgery. In patient education and information consultation, LLMs excel by leveraging natural language generation to provide accurate, comprehensive responses, with some models achieving over 85% accuracy, positioning them as valuable supplementary tools for knowledge dissemination. However, their utility diminishes sharply in scenarios requiring clinical judgment, such as personalized surgical decision-making, where alignment with expert consensus remains low—underscoring that LLMs currently lack the capacity for dynamic reasoning in complex contexts. In clinical practice, LLMs demonstrate supportive potential by enhancing workflow efficiency, automating patient education, and improving information accessibility, particularly in resource-limited settings. They also show promise in medical education, performing well on specialty certification exams. Nevertheless, significant challenges persist, including concerns over information accuracy, timeliness, readability, interpretability, and ethical risks such as bias and data privacy. Future development should focus on establishing real-world evaluation standards, fostering human-AI collaboration, and developing ethical governance frameworks to ensure safe, effective integration into clinical practice  . |
| Limitations | 20 | Discuss the limitations of the scoping review process. | The coverage of the literature included in this study is limited: only Chinese and English literature is included, and important evidence in other languages may be missed. |
| Conclusions | 21 | Provide a general interpretation of the results with respect to the review questions and objectives, as well as potential implications and/or next steps. | Large Language Models have demonstrated potential as auxiliary informational tools and communication mediators in bariatric surgery, showing particular value in enhancing information accessibility, supporting doctor-patient communication, and medical education. However, their current capabilities remain confined to structured knowledge transmission and are not yet reliable for clinical tasks requiring professional judgment, personalized decision-making, and comprehension of complex contexts. Future development should focus on the specialized optimization of models, standardization of evaluation systems, and exploration of clinical integration pathways. This will facilitate the deep integration of AI with bariatric surgery under the premise of ensuring safety, reliability, and equity.  . |
| **FUNDING** | | | |
| Funding | 22 | Describe sources of funding for the included sources of evidence, as well as sources of funding for the scoping review. Describe the role of the funders of the scoping review. | The author did not receive any financial support for the research, authorship, and/or publication of this article. The study received no specific grants from any public, commercial, or nonprofit sector. |

JBI = Joanna Briggs Institute; PRISMA-ScR = Preferred Reporting Items for Systematic reviews and Meta-Analyses extension for Scoping Reviews.

* Where *sources of evidence* (see second footnote) are compiled from, such as bibliographic databases, social media platforms, and Web sites.

† A more inclusive/heterogeneous term used to account for the different types of evidence or data sources (e.g., quantitative and/or qualitative research, expert opinion, and policy documents) that may be eligible in a scoping review as opposed to only studies. This is not to be confused with *information sources* (see first footnote).

‡ The frameworks by Arksey and O’Malley (6) and Levac and colleagues (7) and the JBI guidance (4, 5) refer to the process of data extraction in a scoping review as data charting*.*

§ The process of systematically examining research evidence to assess its validity, results, and relevance before using it to inform a decision. This term is used for items 12 and 19 instead of "risk of bias" (which is more applicable to systematic reviews of interventions) to include and acknowledge the various sources of evidence that may be used in a scoping review (e.g., quantitative and/or qualitative research, expert opinion, and policy document).

*From:* Tricco AC, Lillie E, Zarin W, O'Brien KK, Colquhoun H, Levac D, et al. PRISMA Extension for Scoping Reviews (PRISMAScR): Checklist and Explanation. Ann Intern Med. 2018;169:467–473. [doi: 10.7326/M18-0850](http://annals.org/aim/fullarticle/2700389/prisma-extension-scoping-reviews-prisma-scr-checklist-explanation).
